# Transcriptomic analysis reveals physiological adaptations and immune system modulation in Romanian Holstein cattle during heat stress exposure

**DOI:** 10.1007/s00484-026-03306-1

**Published:** 2026-09-17

**Authors:** Daniela Elena Ilie, Alexandru Eugeniu Mizeranschi, Madalina Mincu-Iorga, Ludovic Toma Cziszter, Ioana Nicolae, Dinu Gavojdian

**Affiliations:** 1 Research Laboratory, Research and Development Station for Bovine, Arad, Romania; 2 Faculty of Bioengineering of Animal Resources, University of Life Sciences, Timisoara, Romania; 3 Institute for Advanced Environmental Research, West University of Timisoara, Timisoara, Romania; 4 Cattle Production Systems Laboratory, Research and Development Institute for Bovine, Balotesti, Romania

**Keywords:** Heat stress, Dairy cattle, Gene expression, Romanian Holstein, Transcriptome analysis

## Abstract

Heat stress represents a significant impairment to productivity, health, and reproductive efficiency of dairy cattle globally. The present study aimed to investigate genes and molecular mechanisms involved in physiological response to heat stress in Romanian Holstein dairy cows, by conducting a whole-blood transcriptomic and bioinformatics analysis. RNA sequencing (RNA-Seq) was performed on samples collected from seven multiparous Romanian Holstein cows under both heat stress (HS, average temperature-humidity index -THI of 77.5) and thermoneutral (TN, average THI of 48.3) conditions. A total of 487 (447 downregulated and 40 upregulated) significantly (|log2FC| ≥ 1 and adjusted p-value ≤ 0.05) differentially expressed genes (DEGs) were identified, during HS exposure compared to TN conditions. Downregulated genes were primarily associated with crucial physiological functions, including immune responses, inflammation, and metabolic pathways, all of which directly influence milk production, health and reproductive efficiency. Upregulated genes revealed active cellular protection mechanisms, notably heat shock proteins (HSPs), and a pronounced activation of the innate immune and antiviral defense responses, alongside metabolic adjustments and complex reproductive responses. Significant enrichment in pathways in response to heat stress included viral defense and lysosomal activity, suggesting an intensified cellular catabolism under thermal stress. In conclusion, Romanian Holstein cattle exhibit a complex, integrated molecular response to heat stress, characterized by a survival-oriented shift in gene expression. These findings provide detailed molecular insights into the physiological challenges faced by dairy cattle during heat stress exposure.

## Introduction

Earth’s temperature has increased per decade since 1850 by an average of 0.06 °C, with a three times higher acceleration rate of warming since 1982, of 0.20 °C per decade. NOAA (2025) reports that 2024 was the warmest year since global records began in 1850, being 1.18 °C above the 20th century average, while alarmingly the 10 warmest years were recorded between 2015 and 2024. The European continent is one of the regions most exposed to climate change, manifested by increases in summer temperatures and the frequency and intensity of heat waves (Vitali et al. 2020; Maggiolino et al. 2022).

Climate change was shown to negatively impact production, reproduction and health, of all livestock species as a result of heat stress (HS) (Lara and Rostagno 2013; Key et al. 2014). However, of all the farmed species the increase in temperature has the largest impact on dairy cattle, which are particularly susceptible to HS as the high yields production generate increased metabolic heat loads (Gauly and Ammer 2020; Cartwright et al. 2023).

Lacetera (2018), Islam et al. (2021), Cartwright et al. (2023) and Messeri et al. (2023) found that HS leads to high risks for metabolic dysfunctions, oxidative stress, and immune suppression in cattle, and consequently negatively alters the animal’s welfare, behaviour, performance and fertility. Therefore, HS poses a significant impediment to sustainable dairy production worldwide, with its prevalence and severity increasing rapidly due to global climate change (Sammad et al. 2020). Dairy cattle experience HS when ambient temperatures exceed 25–26 °C (West 2003) or when the Temperature-Humidity Index (THI) reaches 68 (Brugemann et al. 2012), leading to an inability to dissipate the substantial metabolic and environmental heat loads they accumulate.

To understand the complex molecular mechanisms supporting the physiological responses to thermal stress in dairy cattle, transcriptome analysis, particularly RNA sequencing (RNA-seq), has emerged as a powerful and integral tool (JiaYu et al. 2025). Transcriptomics involves the comprehensive study of gene expression and regulation by quantifying the messenger RNA (mRNA) abundance. This provides a detailed image regarding gene activity within specific tissues under defined conditions, enabling the identification of differentially expressed genes (DEGs), novel gene transcripts, alternative splicing events, and the mapping of complex biological regulatory networks (JiaYu et al. 2025). Recent transcriptomic studies have yielded significant insights into bovine responses to thermal stress, revealing a complex and multi-systemic molecular disruption. For instance, liver transcriptomic analysis in lactating dairy cows has identified hundreds DEGs, with a notable downregulation of mitochondrial protein-coding genes and an upregulation of heat shock proteins (HSPs), indicating compromised mitochondrial integrity and metabolic homeostasis (Li et al. 2023). Blood profiling studies have identified thousands of differentially regulated genes, with enriched pathways related to chaperones, cellular response to heat stress, phosphorylation, immune response, apoptosis, and protein processing in the endoplasmic reticulum (Srikanth et al. 2017). This widespread molecular alterations across diverse pathways indicate that the stress response is a highly interconnected biological event. Studies have shown that even slight increases in core body temperature can cause proteins to become misfolded and can lead to the disruption of cell organelles, which then leads to impaired intercellular transport processes, resulting in the loss of cellular homeostasis and activation of apoptotic cascades (Bellmann et al. 2010; Fulda et al. 2010; Richter et al. 2010). To modulate these pathways and preserve cellular survival, post-transcriptional regulators such as microRNAs (miRNAs) are dynamically altered. For instance, in a study regarding the characterization of miRNA profiles in the mammary tissue of Holstein cattle in response to heat stress, authors identify a number of 27 miRNAs that were significantly differentially expressed between the mammary tissue of heat-stressed dairy cattle and those maintained under normal conditions (Li et al. 2018). Heat stress significantly regulates mammary gland miRNAs, which act as key controllers of critical pathways such as Wnt, TGF-β, MAPK, Notch, and JAK-STAT to mitigate thermal damage (Li et al. 2018).

Despite the significant advancements in transcriptomic studies on thermal stress in dairy cattle, notable knowledge gaps persist. While general genomic regions and variants associated with heat tolerance have been identified, the specific genomic regions involved are still not well documented (Worku et al. 2023). Moreover, there is a distinct absence of specific transcriptomic data for different dairy breeds, given their unique production characteristics and adaptations to local environmental conditions, and as a result the molecular response among breeds to thermal stress may exhibit distinct characteristics.

Selection strategies, such as crossbreeding with thermotolerant breeds and the introgression of the *SLICK1* gene seem to be promising for dairy cattle raised under tropical environments (Carabano et al. 2017). However, such strategies may not viable alternatives for dairy cattle in countries with seasonal variation, which would result in animals that are more susceptible to colder temperatures. In their extensive review paper, Cartwright et al. (2023) found that selecting for physiological and cellular traits represents a promising strategy to introduce thermotolerance in dairy cattle, while minimizing milk yield losses and potential issues with cold stress adaptation. As a result, selecting for high immune response could prove an ideal solution to tackle thermotolerance in dairy cattle raised in both tropical and temperate regions.

Therefore, this study aims to investigate the transcriptome profile of Romanian Holstein dairy cattle during exposure to heat stress, through employing advanced RNA sequencing techniques, seeking to identify differentially expressed genes and molecular pathways involved in thermal stress response.

## Materials and methods

### Ethical statement

All experiments were performed in accordance with relevant guidelines (ARRIVE principles) and regulations (Directive 2010/63/EU on the protection of animals used for scientific purposes). The experimental procedures and protocol were approved by the Ethical Committee of the Research and Development Institute for Bovine (Approval No. 52, issued on July 21 st, 2023).

### Animals, housing, experimental design, and sample collection

This study was conducted at the Experimental Farm of the Research and Development Institute for Bovine in Balotesti, Romania [GPS: 44°37′12″N 26°4′7″E], on Romanian Holstein lactating multiparous dairy cows (parities II and III), between August 2023 and January 2024. Cattle involved in the study-herd had the same feeding regime and management during the experiments and were housed indoors year-round under tie-stall conditions with access to outdoor paddocks (15 m²/head) 10 h per day when temperatures were above 5 °C. Cows were milked twice daily (7 am and 7 pm) and had *ad libitum* access to fresh clean water. Cows’ diets consisted of 7 kg of alfalfa hay, 30 kg of corn silage and 7 kg of concentrates, during both experimental seasons (summer and winter).

In total, seven multiparous Romanian Holstein dairy cows were blood sampled twice, once during heat stress exposure (HS, summer) and once under thermoneutral conditions (TN, winter). Blood samples were collected via the coccygeal vein using Tempus™ Blood RNA Tubes (Applied Biosystems by Thermo Fisher Scientific). After blood collection, all samples were mixed vigorously and stored vertically at −80 °C until RNA isolation. On the day of blood sample collection during the HS period (August 23rd), the maximum temperature reached + 37 °C, the minimum + 23 °C, and the average temperature was + 30.1 °C, with a relative humidity of 44.3% and a temperature-humidity index (THI) of 77.5. During the winter season, when blood samples were collected for thermoneutral conditions (January 12th), the cows were kept exclusively inside the barn, where temperatures ranged between + 5.1 °C and + 11.4 °C, and the average temperature was + 7.4 °C, with a relative humidity of 56.8% and a THI of 48.3, as a result, the winter temperature was considered thermoneutral. The temperature and humidity were recorded using Sencor SWS 12,500 SENC^®^ meteorological station. The THI was calculated according to Kendall et al. (2008): THI=(1.8×T + 32)−(0.55 − 0.0055×RH)×(1.8×T − 26), where T = air temperature (°C) and RH = relative humidity (%).

### RNA extraction, library construction, quality control and sequencing of the transcriptome

Total RNA was isolated from blood samples collected from the same group of animals during both heat stress exposure (HS) and under thermoneutral (TN) conditions, resulting in the collection and analysis of a total of 14 samples. RNA extraction was performed using the TRIzol Reagent (Invitrogen, CA, USA) following the manufacturer’s instructions. Ribosomal RNA (rRNA) was removed from total RNA, followed by ethanol precipitation. RNA integrity and purity were assessed using NanoDrop spectrophotometer (Thermo Fisher Scientific, MA, USA) and a Bioanalyzer 2100 system (Agilent Technologies, CA, USA). Library preparation and sequencing were conducted by Novogene (Cambridge, United Kingdom). Briefly, poly-T oligo-attached magnetic beads were used to isolate the messenger RNA from total RNA. Following fragmentation, random hexamer primers were used to synthesize the first strand of cDNA. The second strand of cDNA was then synthesized using dUTP for strand specific library. The library was assessed using Qubit 2.0 Fluorometer (Thermo Fisher Scientific, USA) and QuantStudio 12 K Flex Real-Time PCR System for quantification and an Agilent 2100 bioanalyzer (Agilent, USA) for size distribution analysis. The qualified libraries were pooled and sequenced on Illumina NovaSeq X Plus platform using a paired-end 150 bp (PE150) sequencing strategy. To guarantee the reliability of the data, quality control (QC) was performed at each step of the library preparation and sequencing procedures.

### Data processing and quality control

Raw FASTQ reads were initially processed using the Fastp software tool (v0.23.4) (Chen et al. 2018). Reads containing adapter sequences, poly-N and low-quality reads were removed from the raw data, yielding high-quality clean reads. Simultaneously, the quality metrics Q20, Q30, and GC content of the clean reads were calculated. All downstream analyses were conducted using these high-quality clean reads.

### Read Mapping to the reference genome

Clean reads were aligned to the reference genome *Bos taurus* (ARS-UCD1.2, Ensembl release v. 111) using HISAT2 (v2.0.5) (Kim et al. 2019). The reference genome and gene annotation files were obtained from Ensembl. A genome index was constructed using HISAT2, which enables accurate mapping through splice-aware alignment, incorporating known splice junctions from the gene model.

### Quantification of gene expression level and differential gene expression analysis

Aligned reads were assembled using StringTie (v1.3.3b) in a reference-guided manner (Pertea et al. 2015). StringTie applies a network flow algorithm and optionally includes de novo assembly to reconstruct full-length transcripts, enabling the detection and quantification of both known and novel splice variants for each gene locus. Gene expression levels were quantified using featureCounts v1.5.0-p3 (Liao et al. 2014), which calculated the number of reads aligned to each gene. Following read counting, differential gene expression analysis between both experimental groups (HS and TN) was conducted using the DESeq2 R package (v1.20.0) (Love et al. 2014). Because the same cows were sampled under both conditions, the DESeq2 analysis was fitted as a paired design, with cow identity included as a blocking factor in the design formula (~ cow + condition), thereby accounting for inter-individual variability while testing the effect of thermal condition. P-values were adjusted using the Benjamini–Hochberg procedure, effectively controlling the false discovery rate (FDR). Genes were considered significantly differentially expressed if they met the criteria of an adjusted p-value ≤ 0.05 and |log2(foldchange)| ≥ 1. Gene expression levels were initially normalized using FPKM (Fragments Per Kilobase of transcript per Million mapped reads) values calculated according to Bray et al. (2016) for quality assessment and visualization. This provided a standardized view of expression distributions across samples, ensuring comparability between TN and HS groups. Pearson correlation coefficients were calculated between all pairs of samples to assess transcriptome similarity and data quality. The correlation matrix was computed using the cor() function in R with the Pearson method, based on log2-transformed FPKM values. Correlation coefficients (R²) were visualized as a heatmap to evaluate within-group and between-group similarities.

Principal Component Analysis (PCA) was used to visualize sample clustering and identify major sources of expression variance between samples. PCA was performed in R on log-transformed expression values, and both two-dimensional (2D) and three-dimensional (3D) PCA plots were generated to visualize clustering of samples and separation between experimental groups. Hierarchical clustering of differentially expressed genes and samples was performed to visualize expression patterns. The clustering heatmap was generated using the pheatmap package in R (v1.0.12). Gene expression values were z-score normalized across samples prior to clustering to enable visual comparison of relative expression patterns.

### Gene ontology (GO) and kyoto encyclopedia of genes and genomes (KEGG) enrichment analysis of differentially expressed genes (DEGs)

Significantly differentially expressed genes were annotated against public databases. Functional enrichment analysis was conducted using clusterProfiler (v4.14.6) (Xu et al. 2024), identifying enriched Gene Ontology (GO) terms and Kyoto Encyclopedia of Genes and Genomes (KEGG) pathways associated with the thermal stress response. Significance tests were performed with the functions enrichGO and enrichKEGG, using default thresholds (0.05 for p-values adjusted by the Benjamini–Hochberg method and 0.2 for q-values). Gene annotations were obtained from the R package org.Bt.eg.db v. 3.20.0 (Carlson 2024).

### Data availability

All raw sequencing data and processed files have been deposited in the NCBI SRA database, under the accession number PRJNA1305363.

## Results

### Sequencing data summary

To investigate the transcriptomic response to heat stress, we performed RNA sequencing on whole blood samples collected from Romanian Holstein dairy cows maintained under two environmental conditions: heat stress (HS, BNR_S_1–7) and thermoneutral conditions (TN, BNR_A_1–7). Across all samples, RNA-Seq generated a total of 1,070,066,390 raw reads, with an average of approximately 76.4 million reads per sample. The data summary is shown in Table 1. The lowest yield was observed for a sample from the TN group with 46,943,376 reads, while the highest was for one of the samples from the HS group, which produced 99,703,394 reads. After quality control and filtering, we retained 1,040,439,906 clean reads, corresponding to an average of 74.3 million reads per sample. The quality metrics of the clean reads indicated high sequencing accuracy, with 97.92% of bases achieving a Q20 score (indicating 99% base call accuracy) and 94.53% achieving a Q30 score (indicating 99.9% accuracy). The average GC content of the clean reads was 45.6%, consistent with expectations for bovine transcriptomes.

**Table 1:** Overview of RNA sequencing data quality in the 14 blood samples

Sample	Raw reads	Clean reads	Error rate	Q20	Q30	GC pct
BNR_S_1	70,452,192	69,328,344	0.01	98.47	95.85	46.66
BNR_S_2	80,990,484	80,515,222	0.01	97.51	93.44	46.52
BNR_S_3	100,163,754	99,703,394	0.01	97.72	93.87	45.66
BNR_S_4	67,543,424	66,006,116	0.01	98.27	95.48	47.07
BNR_S_5	98,777,002	98,146,366	0.01	97.51	93.55	45.8
BNR_S_6	74,991,088	73,256,928	0.01	98.58	96.1	46.05
BNR_S_7	68,241,758	66,908,920	0.01	98.46	95.89	46.79
BNR_A_1	67,845,056	66,356,366	0.01	97.9	94.77	47.89
BNR_A_2	91,074,726	85,135,266	0.01	97.68	93.79	43.3
BNR_A_3	85,671,652	80,725,710	0.01	97.74	93.79	43.02
BNR_A_4	48,376,672	46,943,376	0.01	96.86	92.58	46.95
BNR_A_5	88,370,444	82,467,198	0.01	97.51	93.46	43.8
BNR_A_6	72,238,428	70,801,614	0.01	98.63	96.24	44.28
BNR_A_7	55,329,710	54,145,086	0.01	98.16	94.61	44.67
Total	**1**,**070**,**066**,**390**	**1**,**040**,**439**,**906**				
Average	**76**,**433**,**313.57**	**74**,**317**,**136.14**	**0.01**	**97.92**	**94.53**	**45.60**

### Gene expression level analysis

**Fig. 1:**
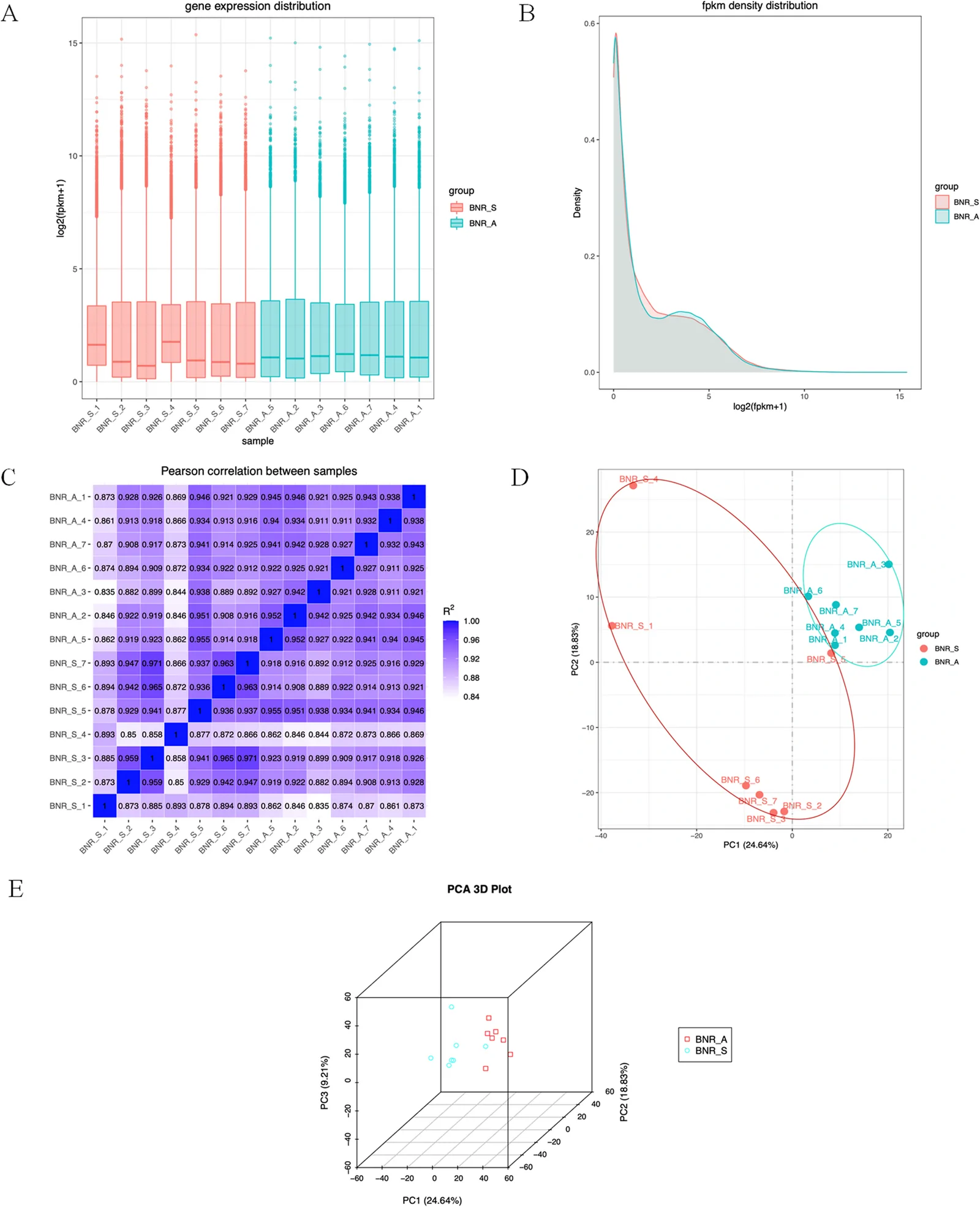
Gene expression level analysis of TH and HS groups. **A**: Gene expression distribution box plot of TH and HS samples **B**: Density distribution diagram of FPKM values. **C**: Pearson correlation between RNA-seq samples (R2: Square of Pearson correlation coefficient). **D**: 2D Principal Component Analysis (PCA) plot. **E**: 3D Principal Component Analysis (PCA) plot

The initial quantification and data processing steps, including the generation of the FPKM (Fragments Per Kilobase of transcript per Million mapped reads) values and the distribution plots, were performed to ensure a standardized analytical pipeline across all investigated samples. To compare gene expression levels under TN and HS conditions, the distribution of gene expression levels and FPKM (Bray et al. 2016) among samples are displayed by boxplots, as shown in Fig. 1A that provide a summary of the central tendency, spread, and skewness of gene expression for each sample. The median log2(FPKM + 1) values demonstrated relative consistency across samples within both groups. The interquartile range was similar across most samples, both within and between groups, indicating that the overall variability in gene expression is largely consistent for the majority of expressed genes across all samples. For the range of gene expression, numerous outliers were observed for all samples, representing genes with higher expression levels. The overall range, indicated by the outliers, extended up to log2(FPKM + 1) values of approximately 15 across all samples, suggesting that the dynamic range of expression captured was substantial and consistent, with no single sample showing a drastically truncated expression profile.

To compare the overall gene expression profiles between the two experimental conditions, the FPKM density distributions for the HS and TN groups were visualized using overlaid kernel density plots of the log2(FPKM + 1) values, as shown in Fig. 1B. Both groups exhibited a broadly similar unimodal distribution shape. For both experimental conditions, the majority of genes were expressed at low levels, with progressively fewer genes detected at higher expression levels. However, the TN group’s distribution displayed a slightly more pronounced broader spread in the mid-range of log2(FPKM + 1) values (approximately from 2.5 to 6). In contrast, the HS group’s distribution is more compressed in this mid-expression region.

Pearson correlation analysis was further performed to assess the relationships between the transcriptomes of all 14 samples (Fig. 1C). The correlation coefficients between samples were generally high, with most values exceeding 0.80. The average correlation among samples in the HS and TN groups was 0.907 and 0.931, respectively. This indicates generally high and consistent similarity within each group. The high correlations observed both within and between groups suggest generally good data quality and consistency across samples.

To further explore the structure of the dataset and identify the major sources of variation in gene expression, Principal Component Analysis (PCA) was performed. The PCA plot (Fig. 1D) demonstrated a clear separation between the HS and TN cattle groups, primarily along PC1, which accounted for 24.64% of the total transcriptomic variance and PC2 explained an additional 18.83% of the variance. Together, the first two principal components account for a cumulative total of 43.47% of the variance. The TN samples form a distinct cluster located in the positive region of the PC1 axis. Conversely, the HS samples form a cluster located almost entirely in the negative region of the PC1 axis. The 3D plot confirms this separation in a higher-dimensional space (Fig. 1E).

**Fig. 2:**
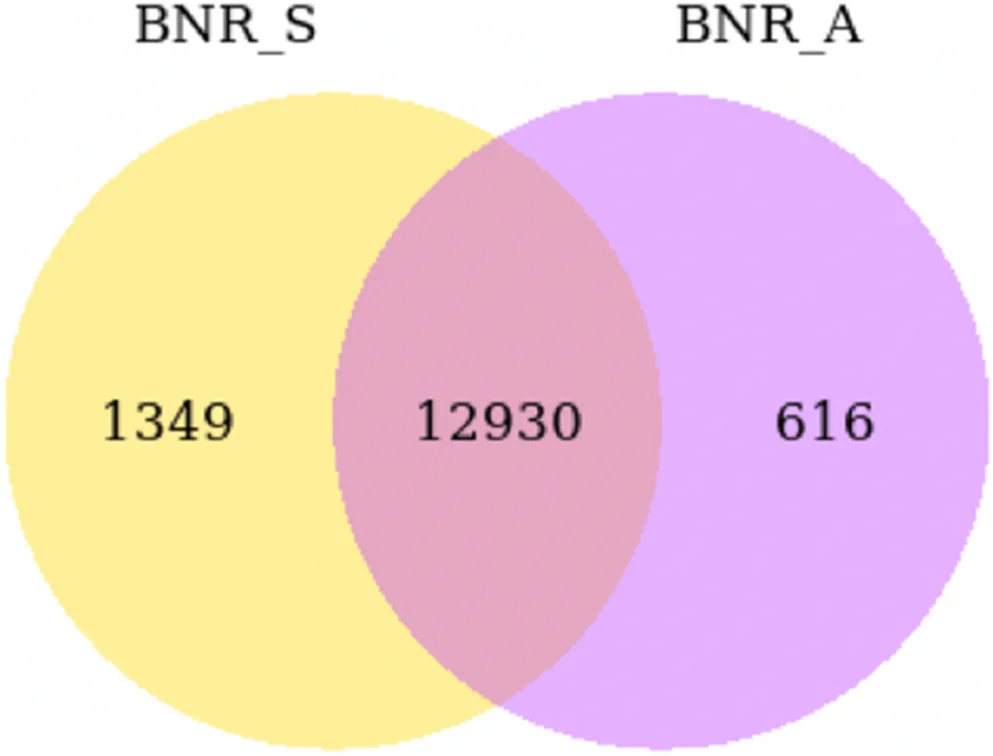
Co-expression Venn diagram during TN (BNR_A) and HS (BNR_S) exposure

The analysis of expressed genes revealed distinct transcriptional profiles for each group. In the TN group, a total of 13,546 distinct genes were identified as expressed. This set comprises 616 genes found exclusively in the TN samples and 12,930 genes that were also expressed under HS conditions. This comprehensive count indicates a broad spectrum of active gene loci under normal physiological temperatures. Under heat stress (HS group), a slightly larger specific transcriptome was observed, with a total of 14,279 distinct genes detected as expressed. This number includes 1,349 genes uniquely expressed in the HS group, in addition to the 12,930 genes shared with the thermoneutral group. The co-expression Venn diagram is presented in Fig. 2.

### Differential gene expression analysis

**Fig. 3:**
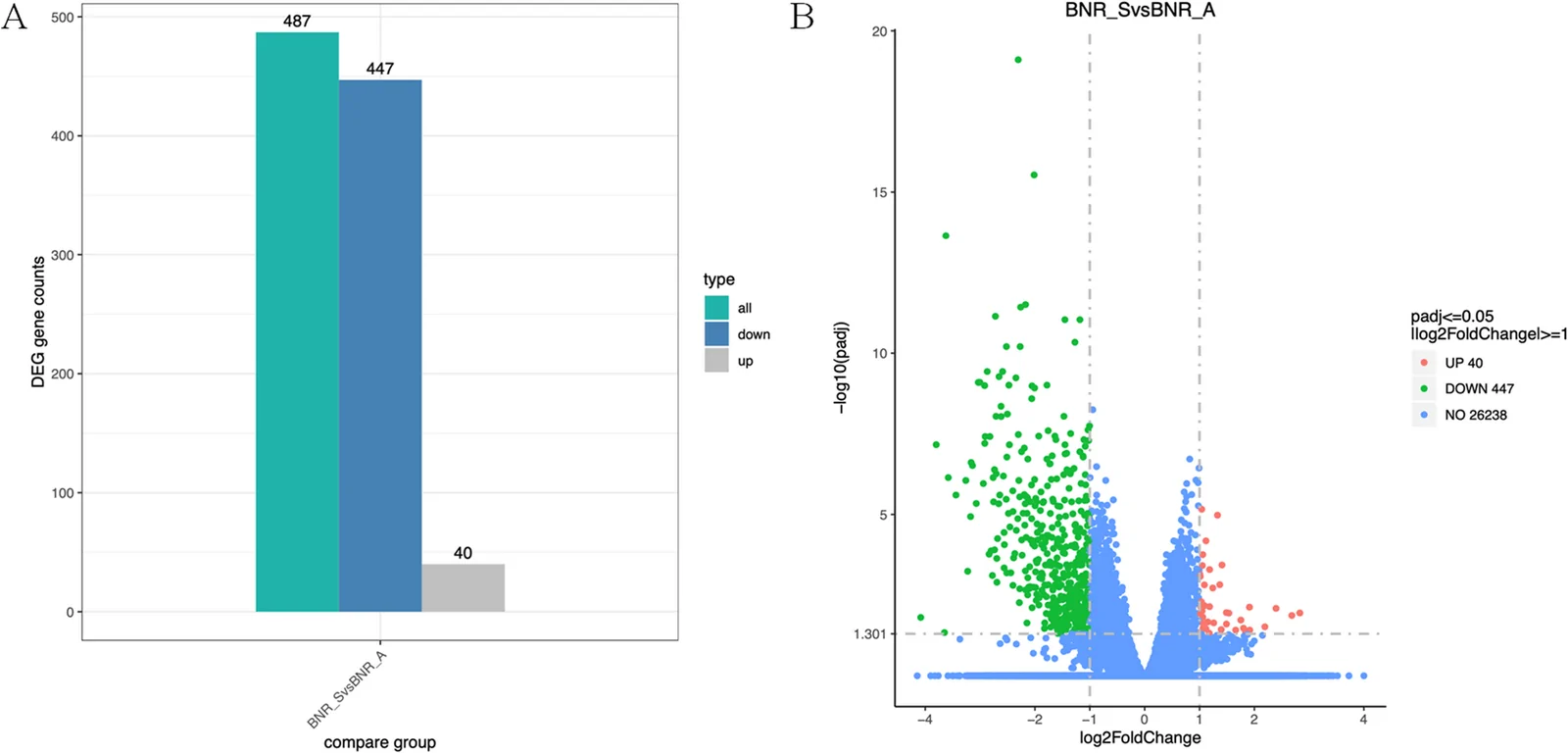
**A**: Differentially expressed genes in HS and TN groups. **B**: Volcano diagram of differential gene expression levels in HS vs. TN group

For differential gene expression analysis, the criteria for significance were an adjusted p-value of ≤ 0.05 and an absolute log2 fold change (∣log 2FC∣) of ≥ 1. Differential gene expression analysis comparing the HS experimental group with the TN control group identified a total of 487 significantly differentially expressed genes (Fig. 3A). A volcano plot was further generated to visualize the magnitude and significance of the transcriptomic changes (Fig. 3B). Of the 487 DEGs, the 447 genes, constituting 91.8% of the total, were significantly downregulated in the HS group relative to the TN controls. The remaining 40 genes, representing 8.2% of the total DEGs, were significantly upregulated. As shown in Fig. 3A-B, there is a high percent of downregulated genes indicating that under the heat stress conditions the gene expression was mainly downregulated.

**Fig. 4:**
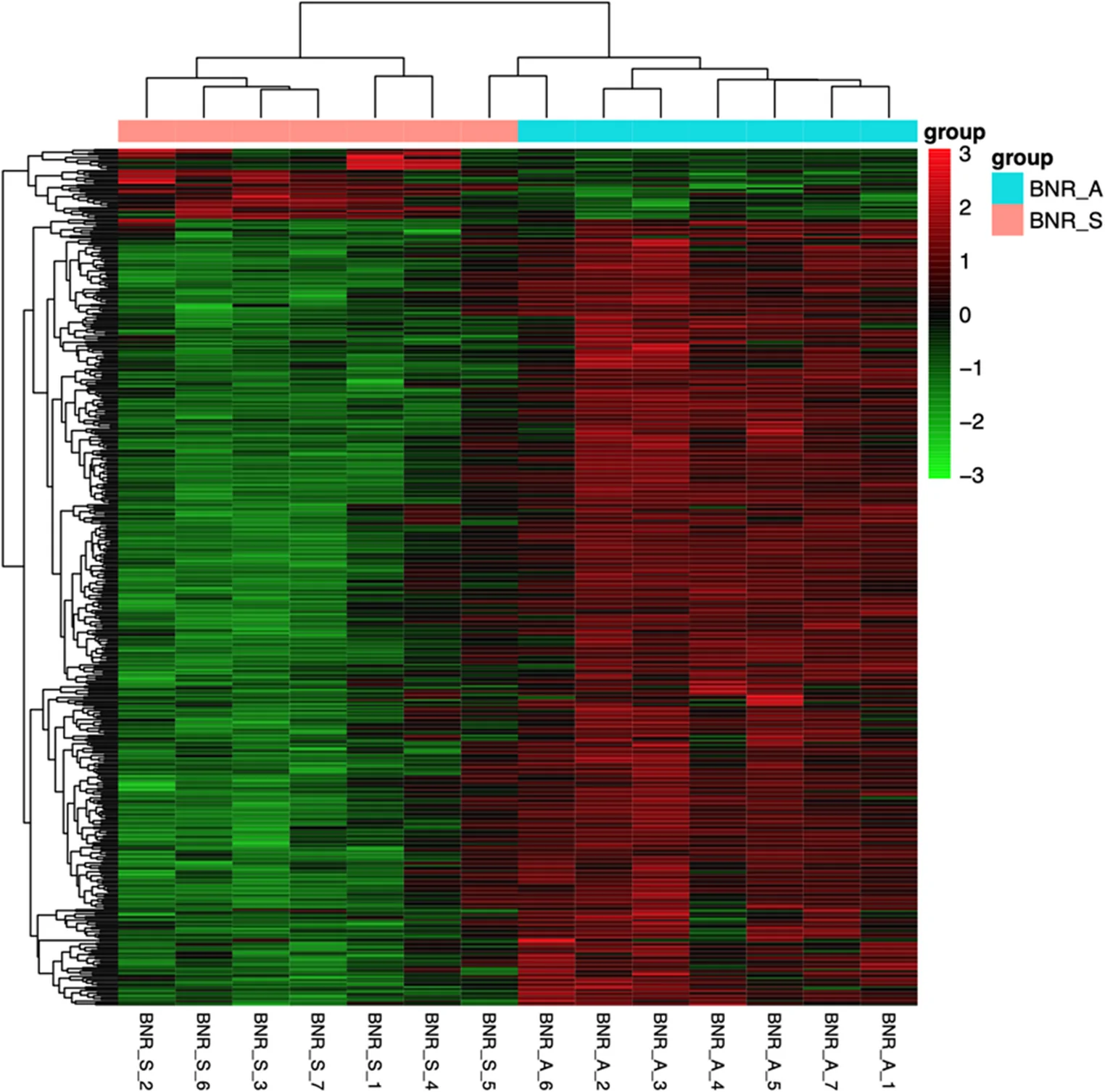
Cluster heatmap of differentially expressed genes during HS and TN exposure

Cluster analysis of DEGs, visualized as a clustering map (heatmap), was performed to explore the expression patterns across all samples and visualize sample relationships. Figure 4 shows the heatmap of clustering among all investigated samples. The heatmap revealed distinct gene expression profiles between the HS experimental group and the TN control group, indicating consistent transcriptional responses within each group and biologically relevant differences gene sets.

### Functional analysis

The set of 487 significantly differentially expressed genes was subjected to enrichment analysis. Gene Ontology enrichment analysis was conducted to identify biological processes (BP), cellular components (CC), and molecular functions (MF) significantly associated with DEGs. To gain insights into the biological functions of the DEGs and reveal specific pathways activated in response to thermal stress in Romanian Holstein cattle, we performed Kyoto Encyclopedia of Genes and Genomes (KEGG) pathway enrichment analyses. The GO and KEGG analysis of the differentially expressed genes in HS vs. TN revealed the involvement of upregulated genes in 10 biological processes, 3 cellular components (Table 2), and 4 KEGG pathways (Table 3).

**Table 2:** Top enriched Gene Ontology terms of DEGs in HS vs. TN

Ontology	ID	Description	Genes	Adjusted *p*-value	Up/Down
BP	GO:0051607	defense response to virus	*OAS1Y*,* RSAD2*,* MX2*	0.001883	up
BP	GO:0009615	response to virus	*OAS1Y*,* RSAD2*,* MX2*	0.001883	up
BP	GO:0140546	defense response to symbiont	*CLEC5A*,* OAS1Y*,* RSAD2*,* MX2*	0.011922	up
BP	GO:0098542	defense response to other organism	*CLEC5A*,* OAS1Y*,* RSAD2*,* MX2*	0.014143	up
BP	GO:0043207	response to external biotic stimulus	*CLEC5A*,* OAS1Y*,* RSAD2*,* MX2*	0.014143	up
BP	GO:0051707	response to other organism	*CLEC5A*,* OAS1Y*,* RSAD2*,* MX2*	0.014143	up
BP	GO:0009607	response to biotic stimulus	*CLEC5A*,* OAS1Y*,* RSAD2*,* MX2*	0.014143	up
BP	GO:0044419	BP involved in interspecies interaction between organisms	*CLEC5A*,* OAS1Y*,* RSAD2*,* MX2*	0.014143	up
BP	GO:0050778	positive regulation of immune response	*C1R*,* CD1E*,* RSAD2*	0.026041	up
BP	GO:0050776	regulation of immune response	*C1R*,* CD1E*,* RSAD2*	0.031023	up
CC	GO:0005764	lysosome	*CTSS*,* CTSB*,* PSAP*	0.005432	up
CC	GO:0000323	lytic vacuole	*CTSS*,* CTSB*,* PSAP*	0.005432	up
CC	GO:0005773	vacuole	*CTSS*,* CTSB*,* PSAP*	0.008546	up

**Table 3:** Top enriched Kyoto Encyclopedia of Genes and Genomes pathways of DEGs in HS vs. TN

ID	Category	Description	Genes	Adjusted *p*-value	Up/Down
bta05164	Infectious disease: viral	Influenza A	*OAS1Y*,* RSAD2*,* IL33*,* MX2*	0.006994	up
bta05171	Infectious disease: viral	Coronavirus disease	*F13A1*,* C1R*,* OAS1Y*,* MX2*	0.014623	up
bta04142	Transport and catabolism	Lysosome	*CTSS*,* CTSB*,* PSAP*	0.014623	up
bta05160	Infectious disease: viral	Hepatitis C	*OAS1Y*,* RSAD2*,* MX2*	0.022817	up

## Discussion

Studies on the transcriptomic response to heat stress in dairy cattle and other ruminants had started to receive a growing interest in recent years (Fang et al. 2021; Hu et al. 2022; Li et al. 2023; Luna-Ramirez et al. 2023; Dige et al. 2024; Halli et al. 2025). In this study we performed RNA-Seq analysis to provide the first whole-blood transcriptomic profile of Romanian Holstein dairy cattle breed under heat stress, characterized by a Temperature-Humidity Index of 77.5. Our analysis reveals a distinct separation of the transcriptome, animals exhibiting different clusters that clearly distinguishes animal under HS from TN condition. This separation is illustrated by the PCA, where the first two principal components account for 43.47% of the total transcriptomic variance, effectively separating the two experimental conditions. These findings are consistent with results from previous studies on livestock transcriptomics, where thermal status has been shown to be a primary driver of gene expression changes, resulting in distinct clustering between heat-stressed and control animals in various breeds (Li et al. 2023; Halli et al. 2025). Moreover, this separation is consistent with several studies demonstrating that heat stress profoundly alters a wide array of biological processes in dairy cattle, including immune function, metabolism, and cellular integrity, leading to a large-scale transcriptomic shift (Yue et al. 2020). The distinct separation observed in our data confirms that the environmental conditions were the cause of transcriptomic variation across the entire dataset that generated biological response at the molecular level across the experimental animals. Our results revealed transcriptional differences between HS and TN condition in Romanian Holstein cattle, with 487 differentially expressed genes identified, including 447 downregulated and 40 upregulated genes in HS compared to TN cattle. Many of the down-regulated genes identified are involved in immune responses, inflammation, and metabolic pathways, all of which directly influence milk production and reproductive efficiency.

The differentially expressed genes involved in thermal stress exposure in Romanian Holstein dairy cows were further explored. The primary biological processes in which some of the down-regulated gene were involved refers to: immune system suppression (*CD200*) (Kotwica-Mojzych et al. 2021); B-cell differentiation (*PAX5*) (Nasri Nasrabadi et al. 2022); TGF-β signaling and tissue development (*SMAD3*) (Zhang et al. 2018); apoptosis and tumor suppression (*WWOX*) (Liu et al. 2018); angiogenesis and inflammation (*RUNX1*) (Bertran et al. 2022), ovarian function and follicular development (*AMHR2*) (Gowkielewicz et al. 2024); growth hormone release and energy metabolism (*GHSR*) (Yin et al. 2014); immune cell differentiation (*BACH2*) (Zwick et al. 2024); bone formation and microtubule organization (*CCDC170*) (Liu et al. 2021); cytoskeletal regulation and organization (*GAS2*,* MACF1*) (Ma et al. 2025; Escobar-Aguirre et al. 2017) and fat metabolism (*CCDC30*) (Eberlein et al. 2010). Dysregulation within these networks, particularly the down-regulation of different key genes, can lead to low performance, increased susceptibility to disease, and significant economic losses in the dairy industry. The consistent down-regulation of genes associated with immune function (*CD200*,* PAX5*,* BACH2*,* ANKRD42*), metabolic health (*SMAD3*,* PPARGC1B*,* NOX1*,* CCDC30*,* LCLAT1*), and reproductive performance (*AMHR2*,* ADAM20*,* CCDC170*) underline the interconnectedness of these physiological systems. The simultaneous down-regulation of some transcription factors (*PAX5*,* BACH2*) involved in important biological processes like immune cell differentiation and function, suggests a coordinated weakening of the adaptive immune system, potentially increasing susceptibility to various infectious diseases.

Heat stress affects multiple microRNAs (miRNAs) which have been recognized as potential biomarkers and key regulators of the stress response (Ren et al. 2025). In addition to the previously mentioned differentially expressed genes, we found down-regulation of multiple miRNAs (*bta-miR-2439*,* bta-miR-326*,* bta-let-7d*) linked to milk production and inflammation (Roudbari et al. 2023; Cendron et al. 2025; Ren et al. 2025), underlining their key role in gene expression. A reduction in their levels could lead to a cascade of dysregulated processes affecting milk quality and the immune response. This indicates that miRNAs are important regulators of complex traits, and their down-regulation can have effects on milk quality and immune health. Besides miRNAs, in the present study, thermal stress exposure in Romanian Holstein dairy cows also leads to the down-regulation of several non-coding RNAs, including the small nuclear RNAs (snRNAs) *U6* and *7SK*, as well as the small nucleolar RNA (snoRNA) *SNORD116*. The down-regulation of *U6* snRNAs, involved in splicing (Parker et al. 2022); *7SK*, key regulator of transcription elongation by controlling the activity of the positive transcription elongation factor b (P-TEFb) (Michels et al. 2003), and *SNORD116*, controlling brain development and the formation of neuronal circuits crucial for managing feeding behavior and maintaining energy homeostasis (Qi et al. 2016), suggests a large impact on gene expression fidelity and metabolic control. A decrease in the expression of these important regulatory RNAs could lead to widespread disruptions in protein synthesis, cellular energy management, and overall physiological balance in dairy cattle, affecting their growth, productivity and resilience to metabolic stressors.

Transcriptomic analyses in Romanian Holstein dairy cows exposed to thermal stress have identified a set of genes that were significantly upregulated, revealing the core biological processes activated to fight cellular damage and maintain homeostasis. The associated biological processes of key upregulated genes in our study refers to: cellular response to heat, protein folding and refolding, maintaining homeostasis (*HSPA1A*) (Fang et al. 2021); regulation of cellular response to heat, transcription factor binding (*HSF1*); inflammation, innate immune response and cell activation (*CLEC5A*); proteolysis, antigen processing and presentation (MHC class II), immune response, extracellular matrix organization (*CTSS*); blood coagulation, fibrin clot formation, and hemostasis (*F13A1*); defense response to bacterium, cell chemotaxis and antimicrobial activity (*DEFB13*); water transmembrane transport, water homeostasis and carbon dioxide transmembrane transport (*AQP1*); complement activation, innate immune response and inflammatory response (*C1R*); DNA-binding transcription factor activity and transcriptional regulation (*ZNF713*); methanethiol oxidation, redox homeostasis and selenium binding (*SELENBP1*); follicle-stimulating hormone signaling, G protein-coupled receptor signaling and gonad development (*FSHR*); pregnancy recognition and placental development, (*PAG19*); innate antiviral response and interferon-gamma-mediated signaling (*OAS1Y*); innate antiviral response, innate immune signaling and lipid metabolism regulation (*RSAD2*); defense response to virus (*MX2*); carbohydrate digestion and alpha-glucosidase activity (SI); regulation of IGF signaling and, MAK cascade (*IGFBP6*); growth, cell population proliferation, IGF1 secretion and protein synthesis (SHE).

The analysis of upregulated genes in Romanian Holstein dairy cows exposed to thermal stress reveals a varied biological response. These findings underscore that cattle employ a complex assembly of molecular mechanisms to manage the detrimental effects of elevated temperatures, impacting cellular, metabolic, immune, and reproductive systems. The observed upregulation of heat shock proteins, such as *HSPH1*, signifies a fundamental cellular commitment to maintaining protein integrity and proteostasis, a critical cytoprotective strategy against heat-induced damage. Simultaneously, the immune system undergoes significant modulation, as evidenced by the increased expression of genes involved in innate immunity (*CLEC5A*, *C1R*), antigen presentation (*CTSS*), and antiviral defense (*OAS1Y*,* MX2*,* RSAD2*). Metabolic adjustments being an important characteristic, with genes like *SI* indicating a compensatory effort to optimize nutrient absorption despite reduced appetite.

The modulation of growth-related pathways, exemplified by *IGFBP6* and *SHE*, suggests a reprioritization of energy allocation, diverting resources from production towards survival and adaptation. Furthermore, the upregulation of *SELENBP1* points to an active engagement of antioxidant defenses to counteract stress-induced oxidative damage. The increased expression of *FSHR* and *PAG19* collectively points to cellular damage within reproductive tissues, compensatory efforts to maintain hormonal sensitivity, and attempts to preserve placental integrity. These findings provide molecular insights into the well documented fertility challenges faced by heat stressed cattle. The upregulation of these genes suggests that animals involved in the study actively struggle to maintain homeostasis under thermal stress. The molecular responses form an integrated network aimed at cellular protection, immune resilience, metabolic adaptation, and reproductive maintenance.

The analysis of DEGs revealed the predominant involvement of upregulated genes with a pronounced enrichment of biological processes primarily involved in immune and defense responses, particularly those directed against viral challenges. Several significant biological process terms were identified, including defense response to viruses (GO:0051607), response to viruses (GO:0009615), defense response to symbiont (GO:0140546), defense response to other organism (GO:0098542), response to external biotic stimulus (GO:0043207), and the response to biotic stimulus (GO:0009607). Genes associated with these viral defense responses include *OAS1Y*, *RSAD2*, and *MX2*, alongside *CLEC5A*. The consistent and highly significant enrichment of multiple response to virus and defense response to virus terms, coupled with the upregulation of these specific genes, points towards a coordinated induction of an interferon-mediated antiviral state as a primary component of the stress response in Romanian Holstein cattle. These genes have in common their induction by interferons and their direct antiviral functions, suggesting that thermal stress in Romanian Holstein cattle may trigger signaling pathways that resemble early viral infection, leading to preparatory antiviral state. This is similar to the research results of Srikanth et al. (2017) which found that calves exposed to heat stress showed significant enrichment in pathways associated with the immune response, specifically, the Toll-like receptor signaling pathway and genes involved in the interferon-related pathways. Moreover, Liu et al. (2020) have reported that in response to heat stress, the immune response system was activated and *OAS2* and *MX2* genes were most significantly enriched in immune effector process.

Beyond specific viral responses, the enrichment of defense response to symbiont (GO:0140546) and response to other organism (GO:0051707) alongside viral responses, with *CLEC5A* as an associated gene, indicates a non-specific innate immune activation. Furthermore, terms such as positive regulation of immune response (GO:0050778) and regulation of immune response (GO:0050776) indicate a broader modulation of the immune system. Genes like *C1R* and *CD1E* were associated with these terms. *C1R* is involved in the innate immune response by triggering phagocytosis, inflammation, and bacterial cell wall rupture and *CD1E* plays an important role in the bovine immune system by presenting lipid antigens to T cells (Van Rhijn et al. 2006). This might indicate that the thermal stress response in Romanian Holstein cattle involves not only immediate antiviral defenses but also the activation of broader immune regulatory mechanisms, including the complement system for rapid pathogen clearance and inflammatory signaling, and specialized antigen presentation pathways, which are important for an adaptive immune response. This suggests that thermal stress triggers both immediate innate responses and mechanisms that can support subsequent adaptive immunity.

The most significant cellular component terms identified were lysosome (GO:0005764), lytic vacuole (GO:0000323), and vacuole (GO:0005773). These terms were associated with genes such as *CTSS*, *CTSB*, and *PSAP*. The enrichment of these terms indicates that organelles are central to the cellular response to thermal stress, serving as critical hubs for degradation and inflammatory signaling. These findings demonstrate that lysosomes are not simply digestive organelles but dynamic structures with diverse functions in immunity (Scott et al. 2025). The upregulation of key lysosomal proteases (*CTSS*,* CTSB*) and lipid-processing proteins (*PSAP*) suggests an intensified cellular catabolism, that imply that cells are actively engaged in clearing cellular debris, potentially from stress-induced damage or metabolic fluctuations (Scott et al. 2025). This points to a critical role for lysosomal function in the thermal stress response of Romanian Holstein cattle.

The most significantly enriched KEGG pathways (bta05164, bta05171, bta04142, bta05160) are predominantly categorized under viral infectious disease, and transport and catabolism. The genes associated with these pathways include *OAS1Y*,* RSAD2*,* MX2*,* IL33*,* F13A1*,* C1R*,* CTSS*,* CTSB*, and *PSAP*. The enrichment of KEGG pathways related to specific viral diseases (influenza A, coronavirus, hepatitis C) suggests that the thermal stress response in Romanian Holstein cattle induces a state of immune readiness that mimics or overlaps with responses to actual viral infections. The associated genes *OAS1Y* and *RSAD2*, are well-known for their antiviral roles. The enrichment of these viral pathways, even in the absence of a known viral infection, suggests that the thermal stress response activates a broad-spectrum antiviral defense. Moreover, the consistent enrichment of the lysosome pathway across both GO and KEGG analyses, coupled with the functions of its associated genes, points to its role in the thermal stress response. This suggests that the thermal stress response in Romanian Holstein cattle involves even an immune preparation at the lysosomal level.

The main limitation of this investigation was the relatively small animal sample size, consisting of seven multiparous Romanian Holstein cows. However, the robustness, precision, and reproducibility of the identified 487 DEGs in this study were heavily preserved through methodological designs. First, the study implemented a fully paired, repeated-measures design where the same seven cows were sampled under both seasonal baselines (thermoneutral and heat stress), with cow identity included in the DESeq2 design formula as a blocking factor, thereby allowing each animal to serve as its own biological control while accounting for baseline inter-individual variability. Second, the statistical framework of DESeq2 is specifically engineered to handle small replicate numbers through empirical Bayes shrinkage (Love et al. 2014). By sharing dispersion information across genes of similar expression strength, DESeq2 stabilizes the log-fold change estimates and corrects for dispersion estimates that are artificially low due to small sample sizes (Love et al. 2014). In the future, we intend to include a higher number of animals, and have a more integrated approach towards studying the effects of heat stress on lactating dairy cattle, such as haematological and biochemical parameters, while following responses during heat, cold and thermoneutral conditions.

The THI values recorded in the present study indicate clearly distinct environmental conditions between the HS and TN periods. The summer HS sample collection was characterized by an average THI of 77.5, driven by an average temperature of + 30.1 °C (reaching a maximum of + 37.0 °C) and a relative humidity of 44.3%. In contrast, the winter TN conditions had an average THI of 48.3, with temperatures ranging from + 5.1 °C to + 11.4 °C and an average relative humidity of 56.8%. Similar THI values have been described in dairy cattle raised under intensive production systems during hot summer periods, where elevated THI was associated with physiological and metabolic alterations. Several studies have reported varying THI threshold values for the onset of heat stress in cattle, with proposed limits generally ranging between 68 and 74 units (Herbut et al. 2018). In northern and temperate climates, production losses for delicate milk components like fat and protein yields begin at extremely low THI thresholds (ranging from 50 to 58), demonstrating that high-yielding animals experience metabolic stress long before clinical symptoms appear (Campos et al. 2022). In intensive tropical dairy production systems, a THI threshold of 76 represents a critical tipping point at which daily milk yield, the milk fat-to-protein ratio, and conception rates undergo severe, concurrent declines (Teawyoneyong et al. 2024). By comparing our values with those of established livestock studies, it is evident that our summer THI of 77.5 represents a state of moderate-to-severe chronic thermal stress, while our winter THI of 48.3 corresponds to thermoneutral conditions and is consistent with values previously reported (West 2003).

There is a growing body of literature showing that heat tolerance in dairy cattle has deteriorated over time, due to negative genetic correlations between milk production and heat tolerance (Brito et al. 2021; Misztal et al. 2025). As a response to challenges of heat stress, the dairy industry has started implementing more frequently management adaptations, such as a more intensive use of cooling fans and water sprinklers. Considering that most of the current genomic selection programs are focused on improving milk yields, which in return acts against heat tolerance, we can assume that improved heat tolerance in dairy cattle can be attained by a stronger emphasis on selection for fitness traits, which in return could act as a proxy for heat tolerance. Within this broader context of integrating both management and genetic approaches to mitigate heat stress impacts, long-term breeding and conservation strategies become essential for maintaining breed sustainability.

Developing long-term genetic conservation and breeding strategies is imperative to maintain the sustainability of the Romanian Holstein breed under the accelerating pressures of global warming. Historically, selection programs have focused almost exclusively on maximizing milk yield, which has created a negative genetic correlation with heat tolerance and increased herd vulnerability to climate-induced heat waves. A valid breeding strategy for Romanian Holstein cattle could be the introgression of tropical adaptations, such as the *SLICK1* gene. However, to build a resilient and sustainable dairy sector, modern breeding strategies must shift toward selecting for heat tolerance by incorporating genomic selection models.

## Conclusions

The RNA-Seq analysis performed in our study revealed significant transcriptional differences in Romanian Holstein cattle under thermal stress. A total of 487 significantly differentially expressed genes were identified between HS and TN dairy cattle. The observed molecular responses in Romanian Holstein cattle under thermal stress represent an integrated network aimed at cellular protection, immune resilience, metabolic adaptation, and reproductive maintenance. This signifies an active struggle by the animals to maintain homeostasis under thermal stress conditions. The contrasting patterns of gene expression, characterized by a predominant downregulation of genes important for reproduction-related functions and complex adaptive immune processes, alongside the upregulation of immediate protective and antiviral responses (heat shock proteins, innate antiviral pathways), suggests a biological prioritization of survival over reproduction and productivity. This indicates a clear biological substitution, where the animal diverts energy and cellular resources from long-term productive functions to immediate survival and cellular repair mechanisms. These findings provide detailed molecular insights into the physiological challenges faced by heat-stressed dairy cattle.
